# Learning to support client autonomy during clinical nursing training: an exploratory qualitative descriptive study

**DOI:** 10.1016/j.ijnsa.2026.100669

**Published:** 2026-09-05

**Authors:** Marjolein M.C.J. Knibbeler, Stan Vluggen, Erik J.L. van Rossum, Sandra M.G. Zwakhalen, Petra M.G. Erkens

**Affiliations:** a Zuyd University of applied sciences, Faculty of Healthcare, Academy of Nursing, Research Centre Community Care, Nieuw Eyckholt 300, 6419 DJ Heerlen, the Netherlands; b Maastricht University, Care and Public Health Research Institute, Department of Health Services Research, Living Lab in Ageing and Long-term Care, Duboisdomein 30, 6229 GT, Maastricht, the Netherlands

**Keywords:** Nursing education, Long-term care, Nursing homes, Community care, Clinical practice, Autonomy-supportive behaviour, Activities of daily living care

## Abstract

**Background:**

Supporting clients’ autonomy in daily living care is vital for dignity and quality of life. In practice, autonomy is often conflated with self-reliance; however, whereas self-reliance concerns performing activities independently, autonomy concerns making decisions according to one’s own values and preferences. Little is known about how nursing students learn to support clients’ autonomy during clinical training.

**Aim:**

We explored how nursing students learn to support clients’ autonomy through interactions with clinical supervisors during clinical training.

**Methods:**

In an exploratory qualitative descriptive study guided by an interpretivist orientation (May–June 2024), we investigated how nursing students learned to support clients' autonomy through 25 non-participant observations in clinical practice and 22 follow-up semi-structured interviews with students (*N = 25*) and clinical supervisors (*N = 21*) across long-term, community, and acute care settings in the southern Netherlands. Data were analysed using a hybrid deductive–inductive thematic analysis.

**Results:**

Autonomy-supportive behaviour was present in practice, although it varied across situations and was not always applied. Supervisors and students often interpreted autonomy support as promoting self-reliance rather than supporting clients' preferences and participation in decision-making. While some supervisors explicitly taught autonomy support through demonstration, feedback, and reflection, these learning opportunities were inconsistent. Consequently, students mainly learned implicitly by observing and imitating supervisors, who acted as role models during everyday care.

**Conclusions:**

Autonomy support was rarely made explicit and was frequently confused with self-reliance. Consequently, students’ learning opportunities were inconsistent and largely implicit. To better support patient autonomy in nursing and community care in the Netherlands, we suggest that supervisors should be supported in making autonomy-supportive practices and their underlying reasoning explicit through structured reflection. In addition, educational programs and practice settings should consider collaborating to develop structured learning opportunities focused on autonomy support.

**What is already known**

• Supporting client autonomy is central to patient-centered nursing care.
• Nursing students typically learn clinical skills through interactions with clinical supervisors in practice.
• Evidence is limited on how students develop autonomy-supportive behaviours during activities of daily living.

**What this paper adds**

• Students primarily acquired autonomy-supportive skills implicitly by observing and imitating supervisors.
• Supervisors often acted as unintentional role models, providing minimal explicit guidance or reflection.
• Structured reflection and collaboration between education and practice may strengthen students’ ability to support client autonomy consistently.

## 1 Introduction

Autonomy – defined as the experience of choice and control over one’s own life, rather than mere independence – is a fundamental human right and a core principle in nursing practice (Ryan and Deci, 2020; Samanta and Samanta, 2005). In practice, autonomy is often conflated with self-reliance; however, these are distinct concepts. Whereas self-reliance refers to the ability to perform activities of daily living or self-care independently, autonomy concerns the right and capacity to make decisions according to one’s own values and preferences (Botana Gronek et al., 2025; Cremer, 2021). Consequently, individuals can exercise autonomy regardless of age or functional ability, making choices that give meaning and value to their lives (World Health Organization, 2017). For older adults or those experiencing functional frailty, nurses play a key role in supporting and enhancing autonomy, balancing individual needs with professional responsibilities while navigating barriers such as institutional routines, time constraints, communication challenges, cultural differences, and family influence (Donnelly and MacEntee, 2016; Govender and Naidoo, 2020; McCormack, 2001). Nursing students develop these skills primarily through clinical experiences, where learning occurs through interpersonal interactions with supervisors and clients.

In most countries, nursing education combines theoretical education in school with clinical training (Berndtsson, 2020; Paunović, 2023). In the Netherlands, national curricula provide foundational knowledge, while clinical placements offer opportunities to apply skills, develop professional attitudes, and practice coping strategies (Eraut, 2000; Knibbeler et al., 2026). Clinical supervisors, as graduated nurses, ideally support the integration of theory and practice. They play a key role by modelling behaviour, facilitating reflection, and guiding students through unpredictable situations by acting as host, mentor, and advisor (Antonsen et al., 2023; Baldwin et al., 2014; Mathisen et al., 2022). Through these interactions, students learn to support client autonomy in activities of daily living, even within constraints such as dependency, risk, and institutional routines (Botana Gronek et al., 2025; Ryan and Deci, 2020).

Clinical training is shaped by European Union directives emphasizing minimum practice hours (European Parliament and Council, 2005/36/EC; 2013/55/EU). Current regulations emphasize the quantity, such as the required 2,300 hours of clinical practice, without addressing the quality and pedagogical aspects of learning (Henriksen et al., 2020). This poses a challenge in optimal supporting clients’ autonomy, which depends on meaningful interactions between students and supervisors that allow students to develop competence and moral sensitivity during autonomy-support (World Health Organization, 2021). However, many supervisors lack formal training in educational guidance, leading to barriers in effective student guidance and teaching behaviour during clinical training (Boakye et al., 2025; Smith et al., 2022; Ulrich et al., 2025). Structural constraints such as workload, time pressure and limited placement availability may hinder students’ opportunities for deep learning (Flott and Linden, 2016). Consequently, learning often occurs informally and implicitly, complicating efforts to understand how autonomy-supportive skills are developed (Berings, 2006).

Researchers have highlighted the importance of role modelling, supervision, and informal workplace learning in nursing education (Jack, 2017; Newton et al., 2009; Vabo, 2021). Role modelling has been found to be an effective way to support learning across both clinical and university settings, with students valuing exposure to both positive and negative role models that help them consider the type of nurse they aspire to become (Jack, 2017). Clinical supervisors play a central role as role models in professional identity development, while supportive clinical placements that provide opportunities for students to gain independence and become part of the nursing team are crucial for workplace learning (Newton et al., 2009; Vabo, 2021). However, it remains unclear how nursing students learn complex implicit competencies, such as supporting clients' autonomy, and whether the established workplace learning processes described above also contribute to the development of autonomy-supportive practices. Although researchers have explored how autonomy support is addressed in theoretical nursing education, they found that autonomy is rarely mentioned in educational documents and that teachers rely on personal experience, leading to inconsistent understanding among students (Knibbeler et al., 2026), the experiential learning that occurs in clinical settings and contributes to the development of autonomy-supportive practices among nursing students remains largely unexplored. We aimed to understand how nursing students learn to support client autonomy through interactions with supervisors, focusing on activities of daily living as key moments for practicing autonomy-supportive behaviour. Activities of daily living are particularly relevant contexts for autonomy-supportive practice because they involve everyday decisions about care, participation, and personal preferences, requiring nurses to continuously balance between providing support, ensuring safety, and meeting overall care expectations while promoting self-reliance and respecting clients’ autonomy (Botana Gronek et al., 2025; Cremer, 2021; den Ouden et al., 2017). In addition, activities of daily living are often experienced by clients as meaningful and purposeful and are important opportunities to maintain or enhance self-reliance. Because these activities are embedded in daily routines and can facilitate participation in other meaningful and social activities, they provide frequent opportunities for nurses and students to support clients’ autonomy in practice (Cremer, de Man-van Ginkel, et al., 2023; Mouchaers et al., 2024). We focused on the following research questions:

1. How does supporting clients’ autonomy occur during clinical training?
2. In what ways do students learn to support clients’ autonomy from their supervisors?

## 2 Methods

### 2.1 Study design

To answer the research questions, an exploratory qualitative descriptive study was employed, guided by an interpretivist orientation (Creswell, 2018). This orientation assumes that participants' experiences and understandings of autonomy support are subjective and shaped by their interactions within the clinical learning environment and was, therefore, viewed as an appropriate research approach. Data was collected in May and June 2024 using two complementary methods: (1) non-participant, direct observations conducted in natural clinical settings, and (2) short follow-up interviews to gain in-depth insights into participants' experiences, intentions, considerations regarding autonomy support, and learning processes.

### 2.2 Setting and sample

The Dutch healthcare sector encompasses various settings, including long-term care (nursing home and community care) and acute care. These settings employ nursing professionals across all Dutch qualification levels, from level 2 Nurse Assistant to level 7 Nurse Practitioner (Table 1). All these certified professionals ideally support clients' autonomy during activities of daily living care according to their level of training.

**Table 1:** Dutch Nursing Qualification Levels (Backhaus, 2017).

Nursing Level	Dutch Qualification Level	Training Length in Years
Nurse Practitioner	7	2
Baccalaureate-Educated Registered Nurse	6	4
Associate Degree Registered Nurse	5	4
Vocationally- Trained Registered Nurse	4	4
Certified Nurse Assistant	3	3
Nurse Assistant	2	2

We focused specifically on interactions between nursing students and clinical supervisors during activities of daily living care in long-term care (nursing homes and community care) and acute care settings in the Netherlands, where autonomy-supportive behaviour is enacted and observed in daily clinical practice. Data were collected across these healthcare settings, involving students from two vocational education and training institutes (levels 2–4) and one university of applied sciences (level 6). Supervisors were qualified nurses (level 4 or 6) responsible for guiding students in activities of daily living care, typically fulfilling roles as mentor, advisor, and role model. Maximum variation sampling aimed for 30 observations across care settings and educational levels (10 per care setting) and included students and supervisors who consented to participate. Student placement data from May–June 2024 was used to identify potential observation locations and initiate contact with relevant healthcare organizations in the southern part of the Netherlands. Once the potential observation locations were known, supervisors (graduated nurses working in direct patient care) and their managers were approached with general study information and asked about their willingness to participate. They were informed that the study aimed to observe everyday collaborative moments between students and supervisors. An everyday collaborative moment was defined as a distinct moment during activities of daily living care in which a student and supervisor interacted around a client-related action, decision, or communication. Multiple moments could occur within a single activity of daily living care observation. To minimize socially desirable behaviour while maintaining informed consent, participants were not explicitly told that the study specifically focused on supporting clients’ autonomy. If the supervisor was interested in participating, the student they were supervising was then asked for consent. Upon agreement from both parties, an observation and semi-structured interview was scheduled

### 2.3 Data collection

All observations took place during activities of daily living morning care (7-9 AM). Morning care was selected because it involves frequent and intensive interactions between students, supervisors and clients, offering multiple opportunities to observe autonomy-supportive behaviour and workplace learning processes. Data were collected by MK and three assistant researchers, using a structured observation guide. The guide was developed based on elements of autonomy-supportive behaviour, aligned with self-determination theory such as “allowing the other to choose the best action” and “fostering interpersonal connections” (Botana Gronek et al., 2025; Ryan and Deci, 2020). In addition, it drew on research about how future nurses are theoretically educated to support clients’ activities of daily living autonomy (Knibbeler et al., 2026), and on Berings (2006) insights on nurses’ on-the-job learning activities. The observations focused on both conscious and unconscious behaviours of students and supervisors during activities of daily living care, with an emphasis on their interaction in the context of developing autonomy-supportive behaviour skills and competencies. Prior to data collection, all researchers were trained in using the structured observation guide to ensure a consistent approach to observing and recording behaviours. Extensive field notes that included both observational notes and reflective notes were taken during the observations by the researchers. The observational notes included description of activities and interaction, and the reflective notes included the researchers’ questions and thoughts while observing. In addition, contextual characteristics of each observation, such as care setting, type of client, and the level of student experience, were documented in the field notes. These contextual factors were considered during analysis to interpret variations in interactions across clinical contexts. During data collection, the researchers held both planned and spontaneous reflexive meetings to discuss observations, contextual influences, and potential researcher bias, as well as to evaluate the observation guide and its practical usability. The observation guide was not adjusted throughout the process, as it served its purpose well. Each researcher was responsible for promptly completing the guide, including detailed notes, using the structured observation guide format. After each observation, a short semi-structured interview was scheduled with both the student and the supervisor to explore perceptions of autonomy-supportive behaviour. In addition, the supervisor’s role in both supporting client autonomy and guiding student learning was discussed, including what supervisors considered important for students to learn in practice and how they themselves acquired the skills to supervise, whether through formal education, workplace experience, or other forms of education. Key findings were documented immediately after each interview using the structured observation guide format (see Supplementary Material, appendix 1 for the observation guide including the interview guide).

### 2.4 Analysis

To enhance rigor and reliability, member checking was offered to participants after the data collection. Data of both the observations and interviews were analysed using a hybrid deductive-inductive thematic approach following the process described by Nowell et al. (2017). The analysis was initially guided by the two research questions and the predefined domains included in the structured observation format, such as interactions between students and supervisors, communication, and factors influencing the learning of autonomy-supportive behaviour. Within this analytical framework, patterns and themes emerging from the data were identified inductively, which aligns with the interpretivist research approach. First, the complete observation reports and interview transcripts were thoroughly read by the first author to become familiar with the data. During this process, recurring patterns, initial reflections, and emerging ideas were highlighted. Subsequently, five randomly selected observation reports and interview transcripts were independently read by two other authors (SV, PE), who also documented recurring patterns, reflections, and ideas. The three authors then discussed the findings and refined the themes by integrating the predefined analytical focus with insights emerging from the data. The first author subsequently coded the data using Atlas.ti 24.0.0.29576 (ATLAS.ti Scientific Software Development GmbH, 2024). Illustrative quotes and excerpts from fieldnotes were selected during the analytic process to exemplify the identified themes and subthemes and to provide contextual insight into participants' experiences. To make the data accessible and manageable for all authors, the codes were summarized in a spreadsheet. Regular meetings ensured alignment across all authors, provided opportunities to discuss uncertainties, and allowed for joint analysis of notable findings (Nowell et al., 2017). In consultation with all authors, it was decided that no additional codes would be added. Data collection and analysis proceeded iteratively until thematic saturation was reached, meaning that no new codes or themes relevant to the research questions emerged from subsequent observations and interviews. This assessment was discussed and confirmed during meetings involving all authors. Several strategies were employed to enhance analytic trustworthiness. Methodological triangulation was achieved by combining observational data with follow-up interviews. The process was systematically documented using structured observation guides, detailed field- and reflexive notes. Reflexivity was fostered through regular discussions in which researchers reflected on their assumptions, interpretations, and potential influence on data collection and analysis. The first author had prior experience in nursing practice and education. To minimize the influence of prior assumptions, interpretations were regularly discussed with the research team and documented in reflexive notes.

### 2.5 Ethical considerations

The Ethics Committee of Zuyderland MC and Zuyd university of applied sciences (METC-Z) reviewed and approved this study (METCZ20220121). To conduct the observations and interviews, both supervisors and students provided written informed consent. Participation was entirely voluntary, and participants could withdraw at any point. Confidentiality was maintained throughout, and ethical approval was obtained from the boards of directors, managers, supervisors and students. Participants were informed that the study focused on observing everyday collaborative moments between students and supervisors. The specific focus on supporting clients’ autonomy was not disclosed prior to data collection to minimize socially-desirable behaviour and preserve the authentic character of the interactions. This partial disclosure involved balancing the aim of observing authentic interactions with the principle of fully informed participation. Given the minimal-risk nature of the study, the ethics committee and the research team considered this approach ethically justifiable and did not withhold information that could reasonably affect participants’ decision to participate. During the observations, informed consent was obtained from the clients involved. The supervisors asked each client for permission before the researcher entered their house or room. After the client consented, the researcher observed quietly within the room. If residents declined, exhibited verbal or non-verbal discomfort, or appeared distressed, the researcher immediately left the premises. Following data collection, a debriefing session was conducted in which participants were informed about the specific focus on autonomy support, the rationale for partial disclosure was explained, and participants were offered the opportunity to withdraw their data, in accordance with established ethical guidelines for research involving partial disclosure (McNallie, 2017).

## 3 Results

Observational and interview data are presented together in the results section, supported by illustrative quotes and fieldnote excerpts. Where relevant, participant characteristics such as educational background, age, and sex are reported.

### 3.1 Background characteristics

A total of 21 supervisors and 25 nursing students participated in the study; see Table 2.

**Table 2:** Participant characteristics.

Characteristic	Supervisors (*N=21*)	Students(*N=25*)
Sex Male Female	4 17	6 19
Mean age (Standard Deviation)	40.38 (SD 14.52)	23.04 (SD 8.58)
Nursing level Nurse aides Certified nurse assistant Vocationally-trained registered nurse Baccalaureate-educated registered nurse	- 6 14 1	2 3 12 8
Year of nursing school 1 2 3 4	- - - -	12 3 4 6

A total of 25 observations took place: 12 in two nursing homes in which clients with somatic diseases resided, six in three community care organizations that included clients with somatic and psychogeriatric care needs, and seven in two acute care organizations, including care and rehabilitation wards.

Within the 25 observations (50 hours), 67 clients were observed, and numerous activities of daily living care interactions unfolded, including dressing, washing, toileting, mobilizing, grooming, and assisting with eating or drinking. The way care was provided varied; either fully taken over, alternately by the supervisor or student, or provided with support, depending on the client’s level of independence. Care took place in different settings, including the client's home (bedroom, bathroom), the client's nursing home room (in the bed, in the bathroom), or the hospital (single/double room).

In eight observations, the student and supervisor worked closely together. In 13 cases, the students worked independently but regularly consulted their supervisor. In these situations, the student was observed, and both the student and the supervisor were interviewed afterwards. In the remaining four cases, no interaction between student and supervisor was observed during the activities of daily living care moments. In two of these four cases, the student worked independently, making direct supervision during activities of daily living care unnecessary, although there was some interaction with the supervisor in the hallway or after the care moment. In the other two cases, the supervisor was absent due to illness or a high workload. Observation data from these four cases were not included in the analysis; however, for the two independent students, follow-up interviews were conducted with both the student and the supervisor. Two supervisors were observed twice, on different occasions and with different students. In total, 22 interviews were conducted. In three cases, interviews could not take place, twice due to high workload and once due to the supervisor being unavailable.

### 3.2 Between supporting and overruling clients’ autonomy

In approximately half of the observed situations, autonomy-supportive behaviour was not observed. In several cases, there was no communication with the client about the care being provided. In other instances, clients’ expressed wishes were not followed. For example, one acute care client was weighed despite explicitly stating she did not want to be. The supervisor took control of the situation without acknowledging the client’s expressed wishes. Moreover, clients were not included in decision-making processes in several observed cases. For instance, in one nursing home, a client who was fully activities of daily living care dependent and bedridden was washed in bed by a nurse. In this situation the client was not offered any choice regarding her care. In the other half of the observations, autonomy-supportive behaviour was demonstrated during activities of daily living care by either the supervisor, the student, or both. In long-term care (nursing home and community care), autonomy-supportive behaviour was demonstrated by “*getting to know the client”* and *“understanding their wishes and needs”*. In long-term care settings, supervisors reported that daily interactions with clients enabled them to get to know clients and their preferences. As one community care supervisor noted: *"I pay attention to autonomy by involving the client and asking about their preferences. It is important to know the client and be able to follow their desired routine."* Supervisors in long-term care reported noticing verbal and non-verbal signals indicating disagreement from the client. Open communication was observed across both long-term- and acute care settings, for example: Supervisors asked open-ended questions about activities of daily living care, such as *"What would you like to do regarding your care?"* and offered choices, for example, about clothing or personal hygiene. When needed, clients were offered options and assisted when making choices, such as in the case of a client with cognitive issues due to dementia. Additionally, in some cases, supervisors demonstrated awareness of their own behaviour. During a home care visit to a client with early-stage dementia who lived independently and tended to mask cognitive difficulties, the nurse initially adopted a directive approach. However, she immediately reflected on this and invited the client to confirm his agreement, thereby involving him in the care process. The nurse stated: *“Sir, we are starting with the pills today. Listen to me, I’m telling you what we’re doing, but do you agree with that?”*

### 3.3 Learning how to support autonomy in everyday practice

To answer the second research question, we explored in what ways students learned to apply autonomy-supportive behaviour during practical learning. During observations in which autonomy-supportive behaviour was present, both explicit and implicit forms of learning were observed, with implicit learning being more frequently observed.

### 3.4 Autonomy support made explicit

Explicit learning was observed when supervisors demonstrated autonomy-supportive behaviour by providing explanations, when students received feedback during task performance, and when active interactions occurred through questioning. During care situations in which autonomy-supportive behaviour was demonstrated, this was often observed through verbal interactions, including asking for and receiving feedback, demonstrating and explaining care practices, and encouraging autonomy through example and reflection.

### 3.5 Demonstration and verbal explanation of autonomy support

In the observed settings, knowledge and experience were seen being exchanged through hands-on demonstration, verbal explanation, and clear instruction. Less experienced students were often seen observing and copying the behaviour modelled by their supervisors. Tasks were first demonstrated and explained, then gradually handed over to the student for independent performance. Students observed supervisors performing tasks, explaining the reasoning behind specific techniques, and giving precise instructions, for example, on how to use aids such as the steady transfer aid or how to properly position a client. Supervisors also reported using this approach deliberately as a teaching strategy. For example, *“I pass this on to students by explaining the situation to clients and setting an example. I let students observe first, after which they gradually take on more of the care themselves. I try to regularly bring up the theme of autonomy during student-supervisor meetings and help the student grow in this area by providing feedback”* (Supervisor, community care). This distinction was also reflected in the observations. Whereas demonstrating focused on how tasks were performed, encouraging through example emphasized supporting client autonomy in practice. During one care moment in a nursing home, the supervisor modelled how to involve the client by first asking for consent before initiating care: Upon entering the room, the client is greeted while still being quite sleepy. The supervisor asks, *“May we assist you with care?”* to which the client agrees. After the client agrees, the supervisor explains to the student “*When Mr. X is having a rough day, we always ask if he wants to be cared for. If he doesn't, we either do it later or not at all. This is always done in consultation with him.”* During several observations, autonomy-supportive behaviour was not explicitly named during care, but it was consistently modelled in the way supervisors interacted with clients. In these cases, supervisors guided students to recognize and support clients’ autonomy within activities of daily living care routines. One supervisor in acute care described how she taught autonomy by integrating small yet meaningful actions into activities of daily living care, such as offering choices or asking open questions. For her, this informal modelling played a key role in student learning, *"Just ask: What works for you? How are things at home? You also look at the bigger picture; how does someone appear? Are they well-groomed or rather not? You can adjust your care accordingly."*

### 3.6 Feedback and reflection

Throughout the observations, students frequently asked for and received feedback. Feedback came from supervisors and occasionally from clients. Less experienced students often received real-time feedback during activities of daily living care, for example, when a client refused food. Supervisors used such moments to explain the importance of respecting client choices. In one case, a community care student provided care to an older man with early-stage dementia who was living independently at home. During the interaction, the supervisor focused on coaching the student, demonstrating such practical skills as how to apply compression stockings. While the student appeared eager and frequently sought non-verbal confirmation from the supervisor, her attention was largely directed toward the supervisor rather than the client. This prompted the supervisor to repeatedly redirect the student’s focus to the client. As the supervisor explained, *“You need to talk to Mr. A […] ask him where he needs help; we cannot make decisions for him.”* Students adjusted their behaviour after receiving client feedback. One final-year nursing home student shared, *“I had dressed the client in clothes they hadn’t chosen, and they became angry. Since then, I always let them decide.”* In another case in a nursing home, a bedridden client with reduced sensation in her lower limbs received care in bed and was, therefore, dependent on assistance. The client expressed a personal preference, asking for powder to be applied under her breasts and explaining where and why this was needed. The student complied and explicitly stated that she would remember this preference for future care moments. In addition, students were seen taking the initiative by asking specific questions about techniques, such as how to attach a catheter or prepare a bed for transfer. Supervisors were observed using these moments to explain and build practical knowledge. During the interviews afterwards, several supervisors talked about their experiences guiding students in learning to support clients’ autonomy, *“As a supervisor, I often supervise students who have mainly worked in long-term care. There, care tasks are more frequently taken over by staff. I then must remind students to let clients do things themselves instead of taking over everything”* (Supervisor, nursing home). During one observation in community care, reflection was explicitly encouraged. A supervisor advised a student not to copy others blindly, *“If someone can do something themselves, ask about it and decide together”* (Supervisor, community care). The student responded: *“I thought everyone was doing it this way, so I just did it too.”* Reflection was also discussed during one interview, where a community care nurse mentioned the value of working together and having regular reflection moments: *“It’s helpful to work together biweekly. I can point things out to her, usually let her handle the care, and observe. We also regularly have reflection meetings to discuss situations”* (Supervisor, community care).

### 3.7 Bringing theory into practice

One student explained how she developed the habit of asking about client preferences and involving them in decisions: “I find it important because I learned about it at school. This topic came up a lot during skills training, especially with my teacher. It was about encouraging patients to do what they are still capable of themselves.”

### 3.8 Autonomy support as an unspoken practice

Implicit learning was observed when supervisors performed care activities without providing explanations of their actions to students. During these situations, students observed the care delivery while the supervisor carried out the tasks. Autonomy-supportive behaviour was present in the interactions between supervisors and clients but was not verbally explained to students. In these contexts, students were present during routine care activities and observed how supervisors performed tasks and interacted with clients during ongoing care processes.

### 3.9 Routine-based learning

During observations, students were seen present during care activities in which supervisors performed tasks without explaining their actions. The observations also showed that students watched supervisors respectfully handle clients who were not actively participating in care. For instance, students observed supervisors handling some situations, like emptying a urine bag or responding when a client refused to eat, without receiving explanations related to supporting autonomy. In one observed situation, the supervisor entered the room, sat down at eye level, and addressed Mr. X. when asking why he did not want to eat. The client responded that he was not hungry. The supervisor then offered an alternative, which the client also refused. The supervisor accepted this refusal. No explanation was provided for the student during this interaction. During the observations of new or less experienced students, it was almost always noted that the supervisor demonstrated tasks, and the students observed. In interviews, supervisors and students mentioned: *“I do this almost automatically. As an individual, I find it important to be involved myself, so I consider it normal to approach the resident in the same way”* (Supervisor, nursing home). *"You're going to take care of someone; they have to agree with it"* (Student, nursing home). “I *stand in the room and hold up two options, 'Would you like this one or that one?' And then I hope the student sees this and picks up on it and takes over"* (Supervisor, long-term care).

### 3.10 Independent practice

More experienced students were almost always observed working independently. According to these students, supervision was more intensive during the first 5 to 14 days, after which they were expected to manage care tasks on their own. One first-year nursing student, who was already a certified nurse assistant, described performing activities of daily living independently and adjusting care based on client responses. He had a meeting with his supervisor every 6 weeks to discuss progress, and, although supervision was available when needed, he performed care tasks independently.

## 4 Discussion

We explored how nursing students learned to support clients’ autonomy during activities of daily living through their interactions with supervisors. Despite autonomy-supportive behaviour being visible in nursing practice, there is often little specific focus on learning about autonomy itself. Our observations suggest that some nurses may not always recognize the autonomy support they provide, as the focus in practice is generally on self-reliance (the client’s ability to perform tasks independently) rather than the ability to make choices in accordance to their needs, which is the essence of autonomy. As a result, much of students’ learning occurs implicitly; they observe and imitate supervisors’ actions without necessarily engaging in critical reflection, which may result in routinized rather than consciously autonomy-supportive practice.

Within the observed cases, autonomy-supportive behaviour was identified in approximately half of the situations, despite its importance in literature as both a fundamental human right and a core principle of nursing practice (Ryan and Deci, 2000; Samanta and Samanta, 2005). Not only is autonomy-supportive behaviour insufficiently demonstrated, but it is also frequently conflated with the concept of self-reliance (Cremer, Vluggen, et al., 2023). However, autonomy and self-reliance are conceptually distinct. While self-reliance refers to the ability to perform tasks independently, autonomy concerns the right and capacity to make one’s own choices. Recognizing the distinction between autonomy and self-reliance is important, as focusing solely on self-reliance may lead to overlooking clients’ preferences, values, and decision-making in care interactions, which are central to providing autonomy-supportive, person-centered care. Supporting autonomy, therefore, requires attention not only to what clients can do independently, but also to how they are involved in decisions about their care. Many supervisors emphasized what clients were still capable of doing independently, framing autonomy in terms of self-sufficiency and linking it to the promotion of self-reliance. This may indicate that supervisors differed to the extent to which they had been explicitly educated on the conceptual distinction between autonomy and self-reliance. However, our data did not provide information on their educational background or training, and, therefore, no conclusions can be drawn regarding the source of these differences. In addition, Botana Gronek et al. (2025) suggested that theoretical frameworks often fail to provide concrete guidance on how to make autonomy-supportive behaviour explicit in daily nursing practice. Moreover, learning to support autonomy appears to be highly dependent on the individual approach and perspective of the supervisor. This pattern aligns with the findings of our previous research into how teachers address autonomy in classroom settings, findings show that the concept of autonomy is rarely mentioned in educational documents and lacks clear guidance (Knibbeler et al., 2026). Teachers rely on personal experience. This pattern suggests that, within the observed context, guidance on supporting client autonomy is experienced by students as variable and largely dependent on individual supervisors’ interpretations, which may contribute to differences in learning experiences across placements (Knibbeler et al., 2026). Based on our findings, we suggest that students often learn how to support clients’ autonomy implicitly through their supervisors. Learning primarily occurs through observation, imitation and participation in activities of daily living care tasks, rather than through explicit instruction. This aligns with the conceptualization of learning as proposed by Eraut (2000), ranging from implicit to explicit, with reactive learning occurring in response to situational demands. In fast-paced healthcare settings, learning frequently emerges as a by-product of action rather than as an articulated educational objective. While such situated learning may be functional in dynamic practice environments, it is not inherently problematic; however, concerns arise when learning involves normative and ethically charged concepts such as autonomy (Dogan and Baykara, 2024). When professional development relies heavily on unspoken routines and copying others, students may reproduce practices without fully understanding the underlying values or intentions guiding their actions. This concern is particularly relevant in activities of daily living care, where supporting client autonomy requires conscious, value-informed decision-making. We suggest that supervisors rarely explicate or verbalize autonomy-supportive behaviour. Instead, they tend to act from personal habits, values, or routines, often without explicit reflection; a pattern consistent with researchers showing that clinical instructors often lack formal preparation for their teaching role and must draw on their individual personal and professional experiences to guide their teaching (Antonsen et al., 2023; Dahlke et al., 2012; Smith et al., 2022). These practices are likely also influenced by broader contextual conditions in clinical settings, such as time pressure and workload (Cusack et al., 2020), which may limit opportunities for explicit reflection and articulation of reasoning. Researchers have documented that time consistently emerges as a limiting condition, with nurses reporting inadequate time available for supervising students (Cusack et al., 2020) and insufficient organizational support for developing explicit teaching competencies (Henderson and Eaton, 2013). Ceelen et al. (2023) emphasized that, in clinical learning, experienced colleagues played a crucial pedagogic role precisely by making such implicit knowledge explicit. Supervisors are positioned as significant others who can support students’ learning by articulating reasoning, values, and professional judgement that students may not acquire independently. Reflection is a key mechanism in this process (Antonsen et al., 2023; Baldwin et al., 2014; Mathisen et al., 2022). Facilitating reflective conversations enables students to critically examine experiences, connects actions to underlying values, and develop intentional professional behaviour (Ceelen et al., 2023; Lucas, 2023). However, we found that reflective questioning by supervisors was rare. As a result, autonomy-supportive skills were predominantly transferred implicitly, which may contribute to routinised rather than deliberately reflected practice in supporting clients’ autonomy in activities of daily living. Within Self-Determination Theory, autonomy-supportive supervision is characterized by practices such as inviting student input, providing rationales, and encouraging reflection, which have been associated with higher-quality learning outcomes (Perlman et al., 2022). In our observations, such explicit practices were infrequent, suggesting that opportunities to actively cultivate student agency through supervision and reflection may be limited in daily practice. In the absence of more frequent reflective dialogue and explicit modelling of reasoning, students’ learning of autonomy-supportive behaviour appears to rely primarily on observation, which may limit opportunities to critically engage with underlying values and intentions.

### 4.1 Strengths and limitations

This study was subject to some strengths and limitations. First, data and methodological triangulation were employed by combining observations with semi-structured interviews of both students and supervisors. This allowed us to verify and deepen our understanding of how students learn to support clients’ autonomy during activities of daily living care. Second, the variation in care settings and participant roles enhanced the credibility and transferability of our findings. This diversity enabled us to identify not only recurring patterns across settings but also to compare similarities and differences between context during the analytical process, thereby strengthening the credibility of the interpretations and supporting transferability to comparable care settings. Third, most students observed were first-year students, which is a particular strength, as it enabled us to examine how autonomy-supportive practices were learned during the early stages of profession socialization. At the same time, this may have limited insight into more advanced or developmental forms or autonomy-supportive practice that could emerge among more experienced students. However, the study also had some limitations. First, the focus on nursing education in the southern part of the Netherlands may limit the transferability of findings to other regions or institutions, due to regional variations in healthcare settings, organization, resources, and client characteristics. Moreover, findings reflect the experiences of nursing students and clinical supervisors within specific long-term care, community care, and acute settings in the Netherlands. Associate degree nursing programmes were not represented in the included settings, which may limit applicability to this educational pathway. Consistent with qualitative research, the aim was to generate rich, context-specific insights that may inform understanding and practice in similar settings. Variations in educational settings may restrict applicability. Second, the duration of the observations was limited, although the study purposefully focused on morning care as a key moment in activities of daily living support. While morning care provided rich opportunities to observe autonomy-supportive interactions, this focus may have captured only specific dimensions of learning and clinical supervision. Valuable learning and supervisory interactions occur throughout different times, including night shifts where unique learning situations, such as report discussions, independent patient assessments, and critical decision-making consultations take place (Campbell et al., 2008; Torwekar et al., 2023). Other learning opportunities, such as care planning discussions, multidisciplinary collaboration, or informal supervisory interactions throughout the day and night, may involve different forms of autonomy-supportive learning that were not observed in this study. Third, there is also a potential risk for observer influence on participants’ behaviour, as the presence of researchers may have affected how students and supervisors acted. Participants may have been more likely to demonstrate communicative behaviours associated with person-centered care, reflection, or autonomy support when being observed. Measures such as establishing trust with participants, limiting disclosure of the study’s specific aim, using a structured observation guide, and follow-up interviews were employed to reduce this risk, but some influence cannot be ruled out. Lastly, it is also possible that additional reflection moments occurred outside the observations. Nevertheless, the interviews were designed to capture the underlying thoughts and reasoning of both students and supervisors as fully as possible. Despite these limitations, we have provided insight into how students learn to support clients’ autonomy during clinical training and how this learning is shaped through interaction with their supervisors during activities of daily living care.

### 4.2 Conclusion and implications

We suggest that Dutch nursing students learn to support clients’ autonomy during activities of daily living care largely through implicit learning processes embedded in interactions with supervisors and everyday clinical practice. Supervisors played an important role in shaping these learning processes, although they were not always aware of their autonomy-supportive behaviour or impact as role models. Given that learning about autonomy support frequently occurred through observation rather than explicit instruction, there may be value in educational initiatives that help make autonomy-supportive practices more visible and discussable. One possible approach is to bring together students, teachers, and supervisors to co-design educational materials that explicitly address clients’ autonomy and how nurses can support it in daily practice. Such materials may also support supervisors in articulating the reasoning underlying their actions and facilitate reflective dialogue in clinical learning environments. By strengthening collaboration between educational institutions and care organizations, these initiatives may contribute to learning processes that are not only experiential but also reflective and intentional. This may help students develop a deeper understanding of how care can be adapted to the individual needs, preferences, and capacities of older adults. As these findings are grounded in specific long-term care, community care, and acute care settings in the Netherlands, they should be interpreted within this context. Further research is needed to explore how autonomy-supportive learning processes are shaped across different educational, organizational, and cultural settings.

## Funding

This research was funded by the Dutch National Fundamentals of Care Programme by The Netherlands Organization for Health Research and Development (ZonMw 80-86300-98-065), The Hague, the Netherlands and Zuyd University of Applied Sciences, Faculty of Healthcare, Academy of Nursing, Research Centre Community Care, Heerlen, The Netherlands, and The Living Lab in Ageing and Long-Term Care and Maastricht University.

## CRediT authorship contribution statement

**Marjolein M.C.J. Knibbeler:** Writing – original draft, Visualization, Validation, Project administration, Methodology, Investigation, Formal analysis, Data curation, Conceptualization. **Stan Vluggen:** Writing – review & editing, Validation, Supervision, Methodology, Formal analysis, Conceptualization. **Erik J.L. van Rossum:** Writing – review & editing, Supervision, Methodology, Conceptualization. **Sandra M.G. Zwakhalen:** Writing – review & editing, Supervision, Methodology, Conceptualization. **Petra M.G. Erkens:** Writing – review & editing, Validation, Supervision, Project administration, Methodology, Funding acquisition, Formal analysis, Conceptualization.

## Declaration of competing interest

The authors declare the following financial interests/personal relationships which may be considered as potential competing interests: Petra Erkens reports financial support was provided by Netherlands Organisation for Health Research and Development. If there are other authors, they declare that they have no known competing financial interests or personal relationships that could have appeared to influence the work reported in this paper.
