# Supplementary material for: Learning to support client autonomy during clinical nursing training: an exploratory qualitative descriptive study
Source: Int J Nurs Stud Adv. 2026 Sep 5;11:100669. doi: 10.1016/j.ijnsa.2026.100669 (PMC13580046; doi:10.1016/j.ijnsa.2026.100669)
Supplement: Supplementary file 1 [file mmc1.docx]

**Observation guide including the interview guide**

**Study:** ………………………………………………………………….

**Organisation:** ………………………………………………………………….

**Location:** ………………………………………………………………….

**Action researcher:** ………………………………………………………………….

**Date:** ………………………………………………………………….

The observations are recorded in as much detail as possible and immediately written up according to a standardised format in line with the checklist, ensuring that nothing is omitted.

*Focus of the observations:*

Activities, events, and behaviours (of both the clinical supervisor and student) during ADL moments, with a focus on the interaction between supervisor and student in the context of learning about autonomy-supportive behaviour.

Observation of facilitating and inhibiting factors related to the interaction between supervisor and student / the learning and teaching of autonomy-supportive behaviour.

Actions that are consciously and/or unconsciously used to teach autonomy-supportive behaviour.

Communication (and manner of communication) between supervisor and student in relation to learning about autonomy-supportive behaviour.

| **Level of actors**  *Who is being observed, what is their professional qualification level, educational level, and year of study? Age, gender? Is the supervisor a “regular nurse” or a nurse who also acts as a clinical supervisor?* |  |
| --- | --- |
| **Care moment and client**  *Which ADL activity/activities are involved? Washing / dressing and undressing / toileting / personal care / indoor mobility / eating and drinking.*  *Which client receives care, and what are the client characteristics (low / medium / high complexity)?* |  |
| **Activities/ events in the context of learning**  *What happens during the learning moment? Who provides care? Does the student observe the supervisor or vice versa? Does the supervisor supplement the student or vice versa? Does either of them ask (in-depth) questions, and if so, which questions? Does one correct the other?* |  |
| **Autonomy-supportive behaviour**  *Do you recognise autonomy-supportive behaviour in the student and/or supervisor (yes/no)? If so, what does it look like? Consider:*   - *Establishing connection by getting to know the person* - *Respecting the person* - *Being aware of one’s own behaviour* - *Facilitating open communication* - *Offering choices* - *Providing support where needed in consultation* |  |
| **Post-observation discussion**  *Optional questions:*   - *Considering the student’s future role, what do you think the student should be taught within this context by the clinical supervisor?* - *What is your role here, and what do you need to teach the student that they do not learn in formal education?* - *How did you learn to supervise students yourself? Was this addressed in your initial education, later in your organisation, or not at all?* |  |
